# Air Pollution and Daily Clinic Visits for Headache in a Subtropical City: Taipei, Taiwan

**DOI:** 10.3390/ijerph120202277

**Published:** 2015-02-17

**Authors:** Hui-Fen Chiu, Yi-Hao Weng, Ya-Wen Chiu, Chun-Yuh Yang

**Affiliations:** 1 Department of Pharmacology, College of Medicine, Kaohsiung Medical University, Kaohsiung 807, Taiwan; E-Mail: chiu358@yahoo.com.tw; 2 Division of Neonatology, Department of Pediatrics, Chang Gung Memorial Hospital, Chang Gung University College of Medicine, Taipei 333, Taiwan; E-Mail: yihaoweng@adm.cgmh.org.tw; 3 Master Program in Global Health and Development, College of Public Health and Nutrition, Taipei Medical University, Taipei 110, Taiwan; E-Mail: bettychiu@tmu.edu.tw; 4 Faculty of Public Health, College of Health Sciences, Kaohsiung Medical University, Kaohsiung 807, Taiwan; 5 Division of Environmental Health and Occupational Medicine, National Health Research Institute, Miaoli 350, Taiwan

**Keywords:** air pollution, headache, case-crossover, clinic visits

## Abstract

This study was undertaken to determine whether there was an association between air pollutant levels and daily clinic visits for headache in Taipei, Taiwan. Daily clinic visits for headache and ambient air pollution data for Taipei were obtained for the period from 2006–2011. The odds ratio of clinic visits for headache was estimated using a case-crossover approach, controlling for weather variables, day of the week, seasonality, and long-term time trends. In the single pollutant models, on warm days (≥23 °C) statistically significant positive associations were found for increased rate of headache occurrence and levels of particulate matter (PM_10_), sulfur dioxide (SO_2_), nitrogen dioxide (NO_2_), carbon monoxide (CO), and ozone (O_3_). On cool days (<23 °C), all pollutants were significantly associated with increased headache visits except SO_2_. For the two-pollutant models, PM_10_, O_3_ and NO_2_ were significant for higher rate of headache visits in combination with each of the other four pollutants on cool days. On warm days, CO remained statistically significant in all two-pollutant models. This study provides evidence that higher levels of ambient air pollutants increase the risk of clinic visits for headache.

## 1. Introduction

Over the past two decades, many epidemiologic studies have shown positive associations between ambient levels of air pollution and increased daily mortality rate [1,2] and hospital admissions or emergency room (ER) visits for cardio-respiratory diseases [3,4,5].

Most epidemiologic studies conducted to date focused on severe events such as mortality, hospitalizations, and emergency department (ED) visits [6,7]. However, more common effects have been less well documented, mainly because of the unavailability of reliable indicators of primary care settings, symptoms, or medication use [8]. A limited number of epidemiologic studies investigated the relationship between air pollutant levels and general practitioner visits for respiratory diseases [9,10,11,12,13]; However, other symptoms have rarely been investigated [8].

Headache is a common clinic problem, an important cause of morbidity in mordern society [14]. A population-based survey reported that 40% of women and 19% of men reported suffering from headache in the past year in Greece [15]. There are many self-reported triggers for headache including weather, food, stress, fatigue, menstruation, and infection [16,17]. The association between air pollution and/or other environmental factors and headache occurrence has not been accepted widely by clinicians [18]. There have been a few studies of the effects of air pollution on headache [8,19,20,21,22,23,24,25,26,27]. However, these results require confirmation and also further explorations using larger datasets.

This study was undertaken to examine the association between levels of ambient air pollutants and clinic visits for headache among individuals residing in Taipei city, the largest metropolitan city in Taiwan, over a 6 year period from 2006–2011, using case-crossover design. Taipei was selected because it is a large city with adequate numbers of outpatient visits, and extensive air pollution data are available. Our results should be applicable to other cities with similar emission sources.

## 2. Materials and Methods

### 2.1. Taipei City

This study examined daily clinic visits for headaches in relation to air pollution levels in Taipei for the 6-year period from 2006 through 2011. Taipei is the largest metropolitan city in Taiwan with a population of about 2.64 million and is located in northern Taiwan. The major air pollution source is automobile exhaust emissions. Taipei has a subtropical climate, with an annual average temperature of 23 °C.

### 2.2. Data Sources Clinic Visits

The National Health Insurance (NHI) Program, which provides compulsory universal health insurance, was implemented in Taiwan on 1 March 1995. Under the NHI, 99% of the island's population receives all forms of health care services including outpatient services, inpatient care, Chinese medicine, dental care, childbirth, physical therapy, preventive health care, home care, and rehabilitation for chronic mental illnesses. In cooperation with the Bureau of NHI, the National Health Research Institute (NHRI) of Taiwan randomly sampled a representative database of 1,000,000 subjects from the entire NHI enrollees by means of a systematic sampling method for research purposes. There were no statistically significant differences in age, gender, and healthcare costs between the sample group and all enrollees, as reported by the NHRI. This dataset (from January 1996 to December 2011) includes all claim data for these 1,000,000 subjects. These database were previously used for epidemiological research, and information on prescription use, diagnoses, and hospitalizations was found to be reliable [28,29].

With strict confidentiality guidelines being closely followed in accordance with personal electronic data protection regulations, the NHRI of Taiwan maintained anonymity and NHI reimbursement data as files suitable for reaearch. In addition, this study was approved by the Ethics Review Board at the Kaohsiung Medical University Hospital (KMUH-IRB-exempt-20130036).

Data on all clinic visits were extracted from medical insurance files for the period 2006–2011. Cases consisted of all patients who had at least one outpatient visit with a primary diagnosis of headache (International Classification of Diseases, 9th revision [ICD-9] code 784.0).

### 2.3. Air Pollution and Meteorological Data

Six air quality monitoring stations were established in Taipei city by the Taiwanese Environmental Protection Administration (EPA), a central governmental agency. The monitoring stations were fully automated and provided daily readings of sulfur dioxide (SO_2_) (by ultraviolet fluorescence), particulate matter (PM_10_) (by beta-ray absorption), nitrogen dioxide (NO_2_) (by ultraviolet fluorescence), carbon monoxide (CO) (by non-dispersive infrared photometry), and ozone (O_3_) (by ultraviolet photometry) levels. For each day, hourly air pollution data were extracted for all of the monitoring stations. After calculating the hourly mean of each pollutant from the six stations, the 24-h average levels of these pollutants were computed. Daily information on mean temperature and mean humidity was provided by the Taipei Observatory of the Central Weather Bureau.

### 2.4. Statistics

Data were analyzed using the case-crossover technique [30,31,32]. This design is an alternative to Poisson time series regression models for studying short-term effects attributed to air pollution [33]. In general, the case-crossover design and time-series approach yielded almost identical results [34,35,36].

A time-stratified approach was employed for the case-crossover analysis [33]. A stratification of time into separate months was made to select referent days as the days falling on the same day of the week within the same month as the index day. Air pollution levels during the case period were compared with exposures occurring on all referent days. This time-stratified referent selection scheme minimizes bias due to non-stationarity of air pollution time-series data [37,38,39]. The results of previous studies indicated that increased hospital admissions or clinical visits were associated with high air pollutant levels on the same day or the previous two days [40]. Longer lag times have rarely been described. Thus the cumulative lag up to two previous days (*i.e.*, the average air pollution levels of the same and previous two days) was used. Because pollutants vary considerably by season, especially O_3_ and PM, and therefore seasonal interactions between air pollutants and hospital admissions or clinic visits have often been reported. However, previous studies were conducted predominantly in countries where climates are substantially different from that in Taipei [41,42,43], which has a subtropical climate with no apparent 4-season cycle. Hence in this study the possible interaction of seasonality on the effects of air pollutants was not considered; but rather temperature was used. The adverse health effects of each air pollutant were examined for the “warm” days (days with a mean temperature above 23 °C) and “cool” days (days with a mean temperature below 23 °C) separately.

The associations between frequency of clinic visits and levels of air contaminants were estimated using the odds ratio (OR) and their 95% confidence intervals (CI) which were generated using conditional logistic regression with weights equal to the number of clinic visits on that day. All statistical analyses were performed using the SAS package (version 9.1; SAS Institute, Inc., Cary, NC, USA). Exposure levels of air pollutants were entered into the models as continuous variables. Meteorologic variables (daily average temperature and humidity on the same day) which might play a confounding role were included in the model. Inclusion of barometric pressure did not markedly change the effect estimates and therefore it was not considered in the final model. ORs were calculated for the interquartile difference (between the 25th and the 75th percentile) of each pollutant, as observed during the study period.

## 3. Results and Discussion

During the six years of the study, there were a total of 81,086 clinic visits for headache in Taipei city. The number of clinic visits for headache and corresponding environmental data are shown in Table 1. There was an average of 37 visits for headache in the city over this study period.

**Table 1:** Daily clinic visits for headaches, weather, and air pollution variables in Taipei, Taiwan, 2006–2011.

Variable ^a^	Min	Percentile			Max	Mean
		25%	50%	75%		
PM_10_ (ug/m^3^)	14.26	34.23	45.79	61.04	888.02	50.67
SO_2_ (ppb)	1.00	2.58	3.51	4.72	11.14	3.79
NO_2_ (ppb)	3.22	19.86	23.65	28.35	61.94	24.44
CO (ppm)	0.13	0.49	0.62	0.78	1.99	0.66
O_3_ (ppb)	4.00	17.92	23.77	30.42	70.89	24.67
Temperature(°C)	9.05	19.33	24.07	28.49	33.18	23.60
Humidity (%)	23.56	66.68	73.13	79.70	94.19	72.86
Headache visits	1	32	40	47	75	37.01

Abbreviation: Min, minimum value; Max, maximum value. ^a^ 24-h average.

Pearson’s correlation coefficients among the air pollutants are presented in Table 2. There was a certain degree of correlation among the pollutants, especially between SO_2_ and PM_10_ (r = 0.46), NO_2_ and SO_2_ (r = 0.52), and between CO and both SO_2_ (r = 0.51) and NO_2_ (r = 0.89).

**Table 2:** Correlation coefficients among air pollutants.

Variable	PM_10_	SO_2_	NO_2_	CO	O_3_
PM_10_	1	0.46	0.37	0.37	0.29
SO_2_	-	1	0.52	0.51	0.07
NO_2_	-	-	1	0.89	−0.06
CO	-	-	-	1	−0.22
O_3_	-	-	-	-	1

The association between various air pollutant levels and number of clinic visits for headache in single-pollutant model are shown in Table 3. For the single pollutant model, number of headache visits were significantly associated with all pollutants on warm days (>23 °C). Significant positive associations were also observed for all pollutants except SO_2_ on cool days (<23 °C).

**Table 3:** Odds ratio (OR) and 95% confidence interval (CI) for daily headache visits for each interquartile range increase ^a^ in single-pollutant model in Taipei, Taiwan, 2006–2011.

Temperature	Pollutant	OR (95% CI) ^b^
≥23° C (*n* = 1222 days)	PM_10_	1.10 (1.08–1.12) *
	SO_2_	1.05 (1.03–1.07) *
	NO_2_	1.15 (1.13–1.17) *
	CO	1.19 (1.17–1.21) *
	O_3_	1.06 (1.04–1.08) *
<23° C (*n* = 969 days)	PM_10_	1.05 (1.04–1.07) *
	SO_2_	0.93 (0.91–0.94)
	NO_2_	1.11 (1.09–1.13) *
	CO	1.04 (1.02–1.06)
	O_3_	1.23 (1.20–1.26) *

* *p* < 0.05; ^a^ Calculated for interquartile range increase of PM_10_ (26.81 μg/m^3^), SO_2_ (2.14 ppb), NO_2_ (8.49 ppb), CO (0.29 ppm), and O_3_ (12.5 ppb). ^b^ Adjusted for temperature and humidity.

Concerns regarding colinearity between air pollutant levels preclude the inclusion of all pollutants into a multiple pollutant model. A two pollutant model was used to gain insight into which individual contaminant might influence the number of headache visits independently of the effects of other pollutants. The results of this analysis are shown in Table 4. It is worthwhile noting that in these analyses in which the effect of a particular pollutant remained significant after each of the other 4 pollutants was included in the model. On warm days, CO remained significantly correlated with headache clinic visits in all the two-pollutant models. NO_2_ remained significant after PM_10_, SO_2_, or O_3_ were included in the model. PM_10_ remained significantly higher after inclusion of SO_2_ and O_3_. SO_2_ remained significant after the inclusion of O_3_. Due to the fact that multiple significance tests were performed, the likelihood of finding a significant negative effect of SO_2_ after inclusion of NO_2_ or CO being due to chance is considerable.

On cool days, PM_10_, O_3_ and NO_2_ were all significant in combination with each of the other 4 pollutants. CO remained significant after inclusion of SO_2_ or O_3_. The effects of SO_2_, however, were significantly negative in the presence of PM_10_, NO_2_, CO, or O_3_. Again the possibility that the finding of a significant negative association is due to chance is considerable.

**Table 4:** Odds Ratio (OR) and 95% Confidence interval (CI) for daily migraine visits for each interquartile range increase ^†^ in single-pollutant model in Taipei, Taiwan, 2006–2011 ^§^.

	Adjusted for PM_10_	Adjusted for SO_2_	Adjusted for NO_2_	Adjusted for CO	Adjusted for O_3_
	OR (95% CI)	OR (95% CI)	OR (95% CI)	OR (95% CI)	OR (95% CI)
PM_10_					
≥23 °C	-	1.10 (1.07–1.12)	1.00 (0.98–1.03)	1.00 (0.98–1.02)	1.09 (1.07–1.12)
<23 °C	-	1.13 (1.11–1.15)	1.02 (1.00–1.04)	1.05 (1.03–1.07)	1.05 (1.04–1.07)
SO_2_					
≥23 °C	1.01 (0.98–1.03)	-	0.93 (0.91–0.96)	0.97 (0.95–0.99)	1.04 (1.02–1.06)
<23 °C	0.84 (0.82–0.87)	-	0.82 (0.80–0.84)	0.85 (0.83–0.88)	0.96 (0.94–0.98)
NO_2_					
≥23 °C	1.15 (1.12–1.17)	1.20 (1.17–1.22)	-	1.03 (0.99–1.07)	1.15 (1.13–1.17)
<23 °C	1.10 (1.08–1.12)	1.22 (1.20–1.25)	-	1.37 (1.32–1.42)	1.26 (1.23–1.29)
CO					
≥23 °C	1.19 (1.16–1.22)	1.21 (1.18–1.23)	1.16 (1.11–1.20)	-	1.19 (1.16–1.21)
<23 °C	1.01 (0.99–1.03)	1.13 (1.10–1.15)	0.79 (0.76–0.82)	-	1.16 (1.14–1.19)
O_3_					
≥23 °C	1.02 (1.00–1.04)	1.05 (1.03–1.07)	1.01 (0.99–1.03)	1.04 (1.02–1.06)	-
<23 °C	1.23 (1.20–1.26)	1.21 (1.18–1.25)	1.44 (1.40–1.49)	1.37 (1.33–1.42)	-

^†^ Calculated for an interquartile range increases of PM_10_ (26.81 μg/m^3^), PM_2.5_ (17.48 μg/m^3^), SO_2_ (2.14 ppb), NO_2_ (8.49 ppb), CO (0.29 ppm), and O_3_ (12.5 ppb). ^§^ Adjusted for temperature and humidity.

This study represents one of few studies on short-term effects of air pollution exposure on number of clinic visits for headache and is the first in Asia. Data showed that the levels of PM_10_, O_3_ and NO_2_ were positively associated with increases in the daily number of headache visits on cool days. For warm days, the most robust associations were found between CO levels and a rise in clinic visits for headache.

An association between air pollution and headache occurrence is biologically plausible. Neurogenic switching, in which exposure to irritants produces an afferent signal that triggers a distant response, potentially in a different organ system, has been hypothesized as a mechanism through which neurogenic inflammation triggered by air pollution exposures may produce migraine headache [44,45]. Alternatively, air pollutants may impair endothelial-dependent vasodialation leading to the development of headaches [46,47]. May [48] proposed a central neurogenic theory supported by neuroimaging where a trigeminovascular reflex may lead to peptide release and subsequent inflammation, vasodilation, and pain.

Women aged 19–27 years who were studied in an experimental chamber, the symptoms of headache, eye irritation, and nasal irritation were significantly worse when exposed to O_3_ at 60–80 ppb compared with less than 2 ppb [49]. Previous studies have demonstrated an association between O_3_ levels and clinic visits for non-migraine headache [8,24]. In Taipei the risk of daily clinic visits for headache also increased with O_3_ concentration on cool days. The mechanisms by which O_3_ may trigger headache are unclear. Future studies are needed to understand the pathogenesis of O_3_ mediated headache.

A daily diary study of headache sufferers in Turin, Italy, revealed that the severity and frequency of headaches was related to numbers of days with increased NO_2_ levels [19]. Our study results further suggested that elevated daily clinic visits for headache are associated with higher NO_2_ levels. This finding is in agreement with previous studies [8,22,23,24,25,26]. The importance of NO_2_ as a cause of increased clinic visits for headache remains unknown. Future analysis of the influence of NO_2_-mediated neural activity and biological plausibility is necessary.

Exposure to CO affects neurogenic inflammation and, consequently, may generate migraine headache attacks [50]. A study conducted on a group of 32 patients displaying various forms of headache in Turin, Italy, revealed a correlation between headaches and exposure to CO [19]. A significant association between elevated CO levels and greater number of headache visits was noted in this study. This is consistent with previous findings [22,23,24,25]. Observed dilation of temporal arteries during migraine headaches and symptomatic improvement with administration of vasoconstrictors suggest a vascular role in headache. Increased pulsation may stimulate stretch receptors and perivascular nerves [51]. Since CO is a well-recognized cardiovascular toxin, a more comprehensive examination of the role of CO in triggerring headache may be worthwhile.

Headache were more commonly reported from a neighborhood with a pulp mill, with higher SO_2_ emissions, compared to one without [20]. The observed effects of SO_2_ were generally not maintained in the presence of other pollutants, and where significance occurred, the effects were in opposite directions from other air pollutants. This finding is not in agreement with previous studies [22,23,24]. The reason for these differences remains unknown. This might be due to collinearity between SO_2_ levels and other pollutant concentrations, which is a common problem in this type of study.

An association between an increased risk for clinical visits for headache and PM_10_ was noted. This finding is not in agreement with previous studies [22,25,26]. The reason for these differences remains unknown. It may be possible that there are differences between Taipei and cities in western countries which include potential differences in susceptibility to PM_10_ between study populations and differences in pollutant toxicities or mixtures. It is also conceivable that the variability in PM_10_ levels may be different due to variability in traffic related pollutants such as CO and NO_2_.

Among the primary air pollutants (O_3_ is a secondary pollutant), NO_2_ and CO appeared to be the pollutants which exert a significant effect on clinic visits for headache. O_3_ is formed from NO_2_ in the presence of hydrocarbons and sunlight in a complex series of reactions. Because the levels of air pollutants are correlated, separating the effects of individual pollutants is problematic. The major source of NO_2_ and CO is from motor vehicle emissions. Thus, motor vehicle emissions may be the responsible source. Because the levels of CO and NO_2_ are highly correlated (*r* = 0.89), separating the effects of CO and NO_2_ is problematic. Whether NO_2_ or CO per se contribute to headache development directly or these gases are simply the best marker of exposure to other toxic pollutants cannot be determined [24].

The case-crossover study design was proposed by Maclure [30] to study the effects of transient, intermittent exposures on the subsequent risk of rare acute-onset events in close temporal proximity to exposure. This design offers the ability to control many confounders by design rather than by statistical modelling. This design is an adaptation of the case-control study in which each case serves as his or her own referent. Therefore time-invariant subject-specific variables such as gender, age, underlying chronic disease, or other individual-level characteristics do not act as confounders. In addition, time-stratified approach [33] was found to be effective in controlling for seasonality, time trends, and chronic and slowly varying potential confounders [37,38,39]. In general, the case-crossover design and the general additive model (GAM) approach, which has been the analytic method of choice for studying the short-term adverse effects of air pollution since 1990 [52], produced almost identical results [34,35,36].

For a factor to confound the relationship between air pollutant levels and headache visits it needs to be correlated with both variables. It is unlikely that smoking and other indoor pollutants confound the present association since day to day variations in indoor emissions, including smoking need not be correlated with outdoor air pollution.

Exposure measurement error is a common concern in environmental epidemiology. Air pollutant levels were assigned from fixed, outdoor monitoring stations to individuals to estimate exposure (assuming that exposure was homogeneous all over the studied area). Exposure measurement errors resulting from differences between the population-average exposure and ambient contaminant levels are not avoidable. However, the potential for misclassification of exposure due to the lack of personal measurements of air pollutant exposure in this study is of the Berkson type and known to produce a bias toward the null and an underestimate of the association [40,53].

Several limitations of the present study need to be noted. First, our study population is homogenous in terms of race compared with populations in other cities and was conducted in a subtropical city. These facts may restrict somewhat the generalizability of these findings to other locations with different meteorological and racial characteristics. Second, behavior such as air conditioning use or time spent outdoors may affect personal exposures. This might affect the magnitude of the observed associations in comparison with other geographic locations. Third, many epidodes of headache do not result in a clinic visit; thus our findings are not generalizable to all such episodes.

## 4. Conclusions

In summary, this study provides evidence of associations between short-term exposure to air pollutants, particularly to PM_10_, NO_2_, CO, and O_3_, and clinic visits for headache. The ecological design of the study precludes the inference of cause and effect. However, these findings reinforce the possible role of air pollution on number of clinic visits for headache.
